# From Paramutation to Paradigm

**DOI:** 10.1371/journal.pgen.1003537

**Published:** 2013-05-23

**Authors:** Ian R. Adams, Richard R. Meehan

**Affiliations:** MRC Human Genetics Unit, MRC Institute of Genetics and Molecular Medicine, University of Edinburgh, Western General Hospital, Edinburgh, United Kingdom; Medical Research Council Human Genetics Unit, United Kingdom

Classical genetic studies aim to understand how genes determine biological processes, physical characteristics, behaviour, and disease by identifying heritable variations in DNA sequence that associate with specific phenotypes. However, organisms ranging from plants to mammals also possess some characteristics that can be inherited in the absence of any causal genetic variant [1], [2]. Examples of such non-Mendelian trans-generational inheritance include the inheritance of prion diseases in yeast [3], trans-generational epistatic interactions modifying susceptibility to germ cell tumours or coat colour in mammals [1], multi-generational transmission of cardio-metabolic disease risk in rats after foetal exposure to glucocorticoids [4], and genotype-independent transmission of parental defence phenotypes induced by herbivore or pathogen attack in plants [5]. Non-Mendelian trans-generational inheritance challenges our understanding of the modes and mechanisms of inherited phenotypic variation, as well as the premise of classical genetic studies in which causal DNA sequence variants are sought within the genome of affected individuals. To date, most of our mechanistic understanding of non-Mendelian trans-generational inheritance comes from studies in plants and nematodes, which use small RNAs to mediate this process, but mechanistic understanding in mammals has been more elusive [2].

Non-Mendelian trans-generational inheritance is linked to epigenetic processes that mediate transmissable changes in gene expression independently of any change in DNA sequence. One specific type of non-Mendelian trans-generational inheritance, paramutation, occurs when one mutant allele induces a heritable epigenetic change in a different allele of the same gene *in trans*, allowing the paramutated allele to cause a phenotype in the absence of any genetic change [1], [6]. Paramutation is best exemplified in plants where there are numerous examples of paramutation inducing phenotypes ranging from differences in pigmentation or morphology to antibiotic sensitivity [6]. Robust examples of paramutation and paramutation-like phenomena have also been described in mammals [7]. In mice, there are mutations in the *Kit* tyrosine kinase receptor that obey the standard Mendelian rules of genetic inheritance and confer pigmentation phenotypes, but engineered mutations in which *LacZ* or GFP are inserted downstream of the *Kit* transcriptional control element can result in *Kit*-mutant pigmentation phenotypes that are inherited and transmitted by genetically wild-type progeny [7]. Interestingly, these paramutagenic *Kit^LacZ^* and *Kit^GFP^* alleles are associated with transmission of aberrant *Kit*-related RNA transcripts to the zygote. Direct microinjection of these aberrant *Kit* RNAs into zygotes is sufficient to induce heritable paramutation-like effects [7]. Microinjection of microRNAs miR-1 or miR-124 into zygotes also appears to induce paramutation-like effects on *Cdk9* or *Sox9*, respectively, resulting in cardiac hypertrophy (miR-1) or embryo overgrowth (miR-124) phenotypes that are transmissable through both the male and female germlines for at least three generations [7].

Although published examples of paramutation are hugely outweighed by examples of classic Mendelian genetic inheritance, understanding the mechanisms that cause paramutation is likely to inform on the way that organisms can establish new heritable epigenetic states during normal development or perhaps in times of nutritional and environmental stress. In plants, the mechanisms that induce epigenetic changes at paramutated alleles are largely unknown, but RNA intermediates and/or physical interaction between the paramutagenic and paramutated alleles have been proposed to initiate paramutation, and enzymes associated with DNA methylation, histone modification, and small RNAs all contribute to this process [6]. In mice, RNA-dependent processes are implicated in paramutation, but the mechanisms that link RNA molecules to heritable epigenetic modification in mammalian systems have not been established. In this issue of *PLOS Genetics*, Kiani and colleagues demonstrate that DNMT2, an evolutionary conserved member of the DNA methyltransferase family, is absolutely required for some types of paramutation in mice: remarkably, paramutation is completely abolished on a *Dnmt2^−/−^* background such that the *Kit^LacZ^* allele is no longer paramutagenic, and miR-124 is unable to induce paramutation-like changes in embryo weight [8].

Although DNMT2 (also known as Trdmt1; tRNA aspartic acid methyltransferase 1) is a member of the DNA methyltransferase family, and can bind and methylate DNA, it is now more closely associated with methylation of RNA, particularly transfer RNAs (tRNAs) [9]. Fission yeast and fruit flies both lack conventional DNA methylation, but encode a DNMT2 homologue that is able to methylate cytosine residues in tRNAs, an activity that is conserved in plants and mammals [9]. Zebrafish require DNMT2 for normal brain, liver, and retina development, but mice, flies, and plants lacking functional DNMT2 are viable, fertile, and morphologically indistinguishable from their wild-type counterparts, despite being unable to modify specific tRNAs [10], [11]. However, fly *Dnmt2^−/−^* mutants exhibit reduced viability under stress conditions and DNMT2-mediated methylation appears to protect tRNAs from stress-induced cleavage in this species [12]. These results strongly suggest a cellular role for DNMT2 in RNA metabolism but, in contrast to the *bone fide* DNA methyltransferases, little evidence has existed that DNMT2 might be able to generate heritable epigenetic phenotypes. The new finding by Kiani and colleagues firmly places DNMT2 at the heart of the mammalian paramutation pathway, and supports previous data suggesting that RNA molecules play a key role in the paramutation mechanism.

**Figure 1:**
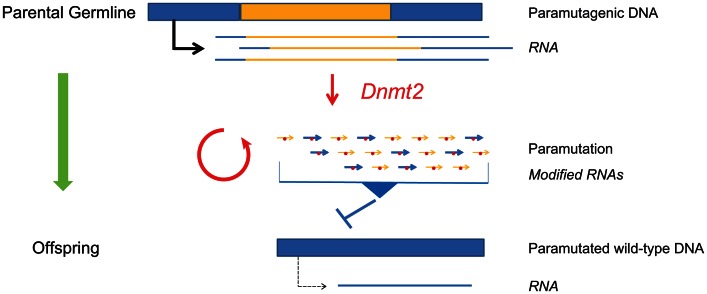
Mammalian paramutation. RNA generated from a paramutagenic allele (blue/yellow) is processed and methylated in a Dnmt2-dependent manner (red circles) in the parental germline, which in turn induces a heritable change in the expression level of the wild-type copy of that allele (blue) in the offspring. The article by Kiani et al. [8] in this issue of *PLOS Genetics* places the RNA methyltransferase Dnmt2 at the heart of the mammalian paramutation mechanism.

A number of potential models present themselves on the basis of these unique observations. Kiani and colleagues [8] demonstrate that at least two tRNAs are methylated in mouse sperm in a *Dnmt2*-dependent manner, which raises the possibility that paramutation transmission is dependent on processed tRNAs. A second model would be that other RNA species, possibly small RNAs, depend on *Dnmt2* activity for their stability, propagation, or subsequent transmission of the paramutated phenotype (Figure 1). The critical requirement for *Dnmt2* in paramutation may be related to the observation in flies that DNMT2-dependent methylation protects RNA molecules against endonucleolytic cleavage. However, injection of *in vitro*–methylated “paramutagenic” miRNAs into *Dnmt2^−/−^* mouse embryos suggests that the role of DNMT2 is not restricted to methylating the injected paramutagenic miRNA, and that the paramutation mechanism involves additional events that we currently know little about. Whatever the case, the dependence of paramutation on Dnmt2 function provides a solid experimental pathway to test cherished hypotheses. This will probably involve massive parallel sequencing of target RNA populations and analysis of RNA modifications in affected and transmitting tissues [13]. It will be exciting to see whether elucidating the mechanisms involved in paramutation will impact our understanding of, and possibly even provide a paradigm for, epigenetic inheritance and RNA-mediated silencing pathways in “normal” biological processes.
